# URIC ACID AND TISSUE REPAIR

**DOI:** 10.1590/S0102-6720201500040018

**Published:** 2015

**Authors:** Rodrigo Araldi NERY, Barbara Stadler KAHLOW, Thelma L SKARE, Fernando Issamu TABUSHI, Adham do Amaral e CASTRO

**Affiliations:** Post-Graduate Program in Principles of Surgery, Evangelic Faculty of Paraná/University Evangelic Hospital of Curitiba/Medical Research Institute, Curitiba, PR, Brazil

**Keywords:** Acid, Cicatrization, Oxygen free radical, Inflammation

## Abstract

Uric acid, a metabolic product of purines, may exert a role in tissue healing. In this review we will explore its role as an alarm initiating the inflammatory process that is necessary for tissue repair, as a scavenger of oxygen free radicals, as a mobilizer of progenitor endothelial cells and as supporter of adaptive immune system.

## Abstract (pt)

O ácido úrico, um produto metabólico das purinas, pode exercer um papel na cicatrização de tecidos. Nesta revisão, será explorado o seu papel no alarme inicial do processo inflamatório que é necessário para o reparo tissular, bem como a sua atuação neutralizadora de radicais livres de oxigênio, mobilizadora de células endoteliais progenitoras e favorecedora da atuação do sistema imunológico adaptativo.

## INTRODUCTION

Tissue damage may occur from a variety of stimuli: infections, trauma, chemical insults, radiation and lack of oxygen and nutrients. Proper healing requires a response where rapid and organized events take place involving several cellular types. Platelets, immune system cells, fibroblasts, endothelial cells and keratinocytes work in a coordinated way to restore homeostasis^15^.

Just after the injury, platelets are engaged in clot formation to limit blood loss and provide protection to the underlying tissues; platelets are also a reservoir for growth factors and cytokines that are released upon degranulation^15^^,^ 25 ^,^ 29. The innate immune system triggers inflammation, promoting a local infiltration of leucocytes whose major role is to kill the invading microorganisms, phagocytize cellular debris and activate keratinocytes and fibroblasts^25^. Sequentially, keratinokytes migrate over the injured dermis and proliferate forming a granulation tissue that intends to restore the barrier function of skin. Fibroblasts invade the clot and angiogenesis occurs. After this, in a slower process, tissue remodeling, commanded by collagen producing fibroblast, leads to scar formation^25^.

 The occurrence of these events requires a coordinated work of a system for detection, containment, and repair of damage caused to cells. This system is composed by warning signals that initiate the process and by cells that respond to them via receptors and signaling pathways^25^. In this system uric acid (UA) seems to play several roles.

Uric acid is generated by the metabolism of purines in most mammals^28^. In lower species, allantoin is degraded by an enzyme called liver uricase resulting in very low serum uric acid levels^28^. However, in humans, a mutation occurred in the evolutionary scale, probably in the late Miocene, making uricase not functional^28^. It is believed that this selection has occurred because of the beneficial effects of uric acid as antioxidant and in the defense against tumor^12^. Furthermore, the UA ability to retain sodium and raise blood pressure could be considered beneficial in situations of food shortage^12^^,^ 28. However, with changing eating habits of the modern diet, rich in salt and uric acid precursors such as fructose, it has been observed that UA is associated with hypertension, coronary artery disease, peripheral vascular disease, renal failure and strokes^8^^,^ 16. Therefore uric acid appears to play a dual role in oxidative stress: antioxidant in the extracellular space and pro-oxidant within the cell^4^^,^ 16. UA is soluble inside cells but precipitates and readily forms monosodium urate (MSU) microcrystals in its extracellular form^13^.

In this review, it will explored UA action in tissue healing.

## THE URIC ACID AS A WARNING SIGN

Our organism must distinguish whether their cells are alive or dead and must be able to detect when microorganisms intrude; so, it can trigger mechanisms of defense and repair. How these mechanisms are activated and orchestrated is still incompletely understood, but we know that a series of dendritic cell receptors are responsible for initiating the process. Some of the best studied receptors are the receptor for PAMPS and DAMPS^13^.

PAMPs or Pathogen-Associated Molecular Patterns are a diverse set of microbial molecules which are shared by several microorganisms which are vital to their survival. PAMPs are recognized primarily through toll-like receptors (TLRs), present in the antigen presenting cell which activates both innate and adaptative immune response^13^. 

When the injury is not caused by a pathogen but by another cause such as trauma, this process is initiated by the alarmins^10^^,^ 13. Thus, alarmin can be considered as the "sterile equivalent of a DAMP". The whole group of alarmins and PAMPS are recognized as DAMPS or Damage Associated Molecular Pattern^13^.

Alarmins are a usually a group of intracellular molecules that are rapidly released following non programmed cell death (necrosis) but not by apoptotic cells^13^. They activate a specific receptor expressing cell (usually a dendritic cell) and recruit the innate immune system, leading to inflammation that is a necessary event to promote tissue reconstruction^10^^,^ 13.

UA is considered a major alarmin released by dying cells^11^. This molecule stimulates the maturation of dendritic cells that trigger inflammation^13^. The increase of serum UA after tissue damage has been shown by Patschan et al.^17^, in a mice model of kidney injury induced by ischemia. They found that, after an ischemic period of 30 min, systemic UA concentration was significantly elevated but restored to normal within 1 h, indicating that this process was rapidly reversible. 

## THE URIC ACID AS SCAVENGER OF REACTIVE OXYGEN SPECIES

Various inflammatory cells like neutrophils, macrophages, endothelial cells and fibroblasts produce reactive oxygen species (ROS) during the process of wound healing^25^. ROS are all oxygen associated species that have higher oxidative potential (higher reactivity) than molecular oxygen. The main members of this group are: oxygen singlet, superoxide anion, hydrogen peroxide and hydroxyl radical (OH-)^25^. 

Oxidants are important in wound healing^21^. ROS play a role potentiating the clotting process; they increase platelet recruitment and collagen induced platelet activation^3^^,^ 9 ^,^ 25. They also affect neutrophil chemotaxis and facilitate the adhesion of neutrophils and monocytes to the extracellular matrix and endothelial cells^14^. ROS help the re-epithelization by activating collagenase expression and mediating EGF (epidermal growth factor) signaling. Furthermore they favor angiogenesis as they enhance the affinity of FGF-2 (fibroblast growth factor) to its receptor^18^^,^ 21 ^,^ 25.

However, oxidants have to be detoxified in order to prevent damage to host cells. If the delicate equilibrium between the produced amount of oxidant and antioxidants system fails, alterations in homeostasis occur that leads to oxidative stress^30^.

Oxidative stress has been demonstrated in chronic wounds such as chronic venous ulcers^30^. Yeoh-Ellerton et al.^30^ have proved that in local fluids from chronic venous ulcer there is high levels of 8-isoprostane that is a prostaglandin-like compound generated by the action of ROS over fatty acids from membrane phospholipids. Oxidative stress prolongs the inflammation and impairs the migration and the synthetic properties of dermal fibroblasts and keratinocytes^25^.

UA is a powerful scavenger of free radicals that provides 60% of free-radical scavenging capacity in plasma^7^. It is considered one of the most prominent antioxidants in the blood of humans and birds^1^^,^ 19. Some studies have shown that there is benefit of intraperitoneal or intravenous administration of UA in experimental models of several disorders that involve increased oxidative stress including multiple sclerosis^5^, Alzheimer's disease^11^, stroke^31^ and spinal cord injury^20^. Unfortunately, UA is relatively insoluble and forms toxic crystals reducing its clinical utility. Chigurupati et al.^191^ developed an UA analog with greater solubility and potent antioxidant activity and have demonstrated that it has a nice effect healing ulcers in animal models.

## URIC ACID MOBILIZING PROGENITOR ENDOTHELIAL CELLS (EPCS)

 It has been shown that UA may accelerate the recruitment of EPCs (endothelial progenitor cells)^17^. In a very elegant study, Patschan et al.^24^, using mice treated with different doses of UA, identified this molecule as a physiologic, fast-acting endogenous mediator of EPC mobilization in response to tissue ischemia. This effect was dependent of dose and duration of the elevation. These authors suggested that UA may be used for pharmacologic preconditioning of EPCs.

## THE URIC ACID AND ITS ROLE IN THE ADAPTATIVE IMMUNE SYSTEM

Depletion of uric acid by allopurinol reduces the generation of immunity to transplanted cell antigens^24^. When UA is co-injected with antigen in vivo, it significantly enhances the generation of responses of CD8+ T cells^23^. It is believed that uric acid leads to an increased T cell response because of its role in the activation of presenting antigen cells^23^^,^ 24.

Lymphocytes play a crucial role in tumour defense by inducing cytotoxic cell death and inhibiting tumour cell proliferation and migration^2^. Regarding these findings, an elevated level of UA should be associated with better prognosis in cancer, which was indeed be found by Dziaman et al.^6^. They showed that survival time in colorectal cancer patients is higher in those with higher UA serum levels. Also, it has been displayed that elevated serum UA levels may protect against cancer mortality in a large study of 1.823 males with lung, colorectal and prostate cancer^27^. Nevertheless, this tumor protecting action of uric acid has not been accepted by others^26^.

## CONCLUSIONS

There are several gaps in our knowledge of the UA role in the microenvironment of tissue wounds. Despite this, there are some clear suggestions that pursuing the study of the inflammatory and immunological role of this molecule may offer new ways of understanding the basis of tissue healing and how to manipulate it for the benefit of patients.
